# Experimental Study on Crack Evolution Law of a Full-Scale Prestressed Concrete Beam Based on Fractal Theory

**DOI:** 10.3390/ma19143129

**Published:** 2026-07-21

**Authors:** Zhenyu Jiang, Bo Wang, Hongyi Liu, Jinquan Zhang, Jianting Zhou, Haifang He, Han Wei, Jingyan Zou

**Affiliations:** 1 School of Civil Engineering, Beijing Jiaotong University, Beijing 100044, China; 2 China-Road Transportation Verification & Inspection Hi-Tech Co., Ltd., Beijing 100088, China; hongyi.liu@ctvic.cn (H.L.); jyzou@chd.edu.cn (J.Z.); 3 Anhui Province Highway Management Service Center, Hefei 230041, China; bowang116@163.com; 4 Research Institute of Highway Ministry of Transport, Beijing 100088, China; jq.zhang@rioh.cn (J.Z.); hf.he@rioh.cn (H.H.); h.wei@rioh.cn (H.W.); 5 School of Civil Engineering, Chongqing Jiaotong University, Chongqing 400074, China; jtzhou@cqjtu.edu.cn

**Keywords:** bridge engineering, PC beam, full-scale experiment, crack, fractal theory

## Abstract

There are different degrees of cracks in prestressed concrete (PC) beams, especially the transverse cracks in the bottom plate and the vertical cracks in the web of prestressed concrete box girders, which will reduce the bearing capacity of the structure. In order to clarify the evolution law of cracks in prestressed concrete box girders after cracking, a 25 m prestressed concrete box girder was poured and a full-scale model test was carried out. At the 1/4 span position of the test beam, the test beam was cracked in 19 steps to 1900 kN. Then at the 3/4 span position, the test beam was cracked in 18 steps to 1800 kN, and after that the bending load test was carried out in 17 steps to analyze the influence of existing cracks on the bending cracks of the test beam. Combined with fractal theory, the evolution law of cracks, the development law of fractal dimensions, and their relationship under different loading conditions were analyzed. The results showed that the crack distribution of the test beam satisfies self-similarity and had fractal characteristics in a statistical sense, which could reflect the process of crack occurrence, propagation and damage, and could quantify the crack shape. The fractal dimension of cracks gradually increased with the increase in concentrated load on the test beam, and the fractal dimension of cracks had a linear relationship with the concentrated load. The crack propagation process of the concrete beam reflected the fractal dimension increasing process; that is, the crack damage evolution process of test beam was a dimension increasing process, and the fractal dimension of crack distribution increased linearly with the applied load. The functional relationship between prestressed concrete crack width and fractal dimension was constructed, which provided a new path for bridge safety evaluation. The research results provide engineering reference for the cause analysis and maintenance treatment of similar bridge cracks.

## 1. Introduction

Prestressed concrete (PC)box girders have been extensively employed in long-span bridge structures owing to their superior structural efficiency, spanning capability, and durability [1,2,3,4]. Nevertheless, during prolonged service life, these structures are inevitably subjected to coupled mechanical loading and environmental actions, resulting in the initiation and progressive propagation of cracks. Cracking represents one of the most critical distress modes in PC girders, directly compromising structural stiffness, load-bearing capacity, and long-term durability [4,5,6,7]. Conventional crack characterization approaches predominantly rely on macroscopic geometric indices such as crack width, spacing, and count [8]. However, these metrics often fail to capture the inherent complexity, irregularity, and multi-scale nature of evolving crack networks [9,10]. Consequently, there is an urgent demand for advanced quantitative methodologies capable of comprehensively describing the evolution law of cracks in full-scale PC structures.

Fractal theory provides a powerful mathematical framework for quantifying geometric irregularity and self-similarity in natural phenomena and has been increasingly recognized as an effective tool for characterizing crack patterns [11]. Fractal theory converts the geometric irregularity of cracks into computable mathematical parameters through the Fractal Dimension (hereinafter referred to as FD). Early investigations predominantly employed monofractal analysis—particularly the box-counting dimension—to describe the overall complexity of crack networks. Ebrahimkhanlou et al. [12] conducted pioneering fractal and multifractal analyses of crack patterns in prestressed concrete girders, demonstrating that fractal dimensions can effectively distinguish crack characteristics under varying loading conditions. Subsequent studies extended this approach to diverse structural contexts. Liu et al. [13] analyzed the crack morphology and propagation behavior of steel fiber geopolymer lightweight aggregate concrete beams. Zhao et al. [14] analyzed the flexural performance and crack development law of engineered fiber-reinforced cementitious composite concrete composite beams. Luan et al. [15] investigated flexural performance of CFRP-strengthened concrete beams. Tang et al. [16] analyzed the flexural cracks in prestressed concrete girder bridges, and Dai et al. [5] explored fractal characteristics of cracking processes in corroded prestressed concrete beams. These investigations collectively established the foundational role of fractal geometry in concrete crack analysis.

Despite these advances, monofractal dimensions inherently assume spatial uniformity of crack distributions and are insufficient to characterize the heterogeneous, multi-scale features of complex crack networks [10]. In recent years, the research paradigm has progressively shifted toward multifractal analysis, which offers a continuous spectrum of scaling exponents capable of capturing local singularities and non-uniformity within crack patterns. Wang et al. [17,18] developed multifractal analytical methods and investigated the multifractal characteristics of fatigue cracks in full-scale reinforced concrete hollow-slab beams, demonstrating that multifractal parameters exhibit superior sensitivity to crack evolution stages compared to monofractal dimensions. Li et al. [19] applied multifractal spectrum analysis to characterize the evolution of multiple cracks in concrete beams, while Pan et al. [10] introduced multifractal-spectrum shape parameters to quantify the distribution and evolution of multiple cracks in concrete structures. Furthermore, Wang et al. [20] systematically examined the effects of scale parameters and counting origins on box-counting fractal dimensions, providing critical methodological guidance for standardized crack analysis. This trajectory reflects a clear developmental trend from global, single-scale descriptors toward localized, multi-scale quantitative characterization of concrete cracking behavior.

Parallel to the advancement of fractal theory, full-scale experimental investigations have become essential for understanding the authentic mechanical behavior of PC structures, as scaled models often fail to replicate the complex stress redistributions and crack propagation patterns observed in prototype structures [2,3,21]. Fang et al. [2] conducted full-scale model tests on the mechanical performance of prestressed concrete simply supported box girders, while Zhou et al. [3] performed full-scale tests focusing on stiffness degradation and damage identification of prefabricated PC box girders. Liu et al. [21] utilized full-scale bending tests to investigate crack variation in PC hollow core beams, and Yu et al. [4] studied the residual bearing capacity of damaged prestressed concrete box girders. Zeng et al. [7] further examined the flexural performance of post-tensioned prestressed concrete T-beams under local corrosion. Despite these valuable contributions, existing full-scale studies have predominantly concentrated on macroscopic mechanical responses—such as bearing capacity, stiffness degradation, and failure modes—while systematic quantitative characterization of crack network evolution using fractal or multifractal theory remains notably scarce.

Moreover, the integration of fractal analysis with advanced image processing and intelligent algorithms represents a significant contemporary trend in structural health monitoring. Liu et al. [9] demonstrated that structural performance assessment of concrete components can be effectively conducted based on fractal information extracted from crack patterns. Recent investigations have incorporated graph theory [22], deep learning [23], and machine learning [24,25] for automated damage assessment utilizing crack pattern features. Concurrently, technological innovations in crack data acquisition—including multi-scale robotic measurement systems [26] and image complexity quantification methods [27]—have substantially enhanced the precision and efficiency of crack documentation. Li et al. [28] combined two-dimensional image analysis with fractal theory for quantitative evaluation of impact cracks, illustrating the synergistic potential of digital image processing and fractal characterization. These developments collectively underscore an evolving paradigm toward intelligent, automated, and multi-scale crack monitoring frameworks.

Despite the aforementioned progress, several critical gaps persist in the existing literature. First, although fractal and multifractal analyses have been applied to various concrete members, comprehensive studies focusing specifically on crack evolution in full-scale PC box girders under flexural loading are limited. Second, the quantitative correlation between fractal/multifractal parameters and the mechanical degradation (e.g., stiffness reduction, damage accumulation) of full-scale PC beams has not been systematically established. Third, the physical significance of multifractal spectrum parameters in characterizing distinct stages of crack initiation, propagation, and coalescence in large-scale PC structures requires further clarification.

To address these gaps, this study presents an experimental investigation into the crack evolution law of full-scale prestressed concrete box girders based on fractal theory. The primary contributions of this research are threefold: (1) A full-scale bending test was conducted on PC box girders, and high-resolution crack images were acquired throughout the entire loading process, enabling detailed documentation of crack initiation, propagation, and network formation. (2) Both monofractal and multifractal analyses were employed to quantitatively characterize the evolution of crack networks, and the sensitivity of various fractal parameters to different damage stages was systematically evaluated. (3) The correlation between fractal/multifractal descriptors and structural stiffness degradation was established, providing a novel quantitative framework for damage assessment and remaining service life prediction of PC girder bridges. The findings of this study are expected to advance the understanding of crack evolution mechanisms in full-scale PC structures and offer practical tools for structural health monitoring and maintenance decision-making.

## 2. Full-Scale Experimental Study on PC Beams

### 2.1. Overview of the Test Beam

**Figure 1:**
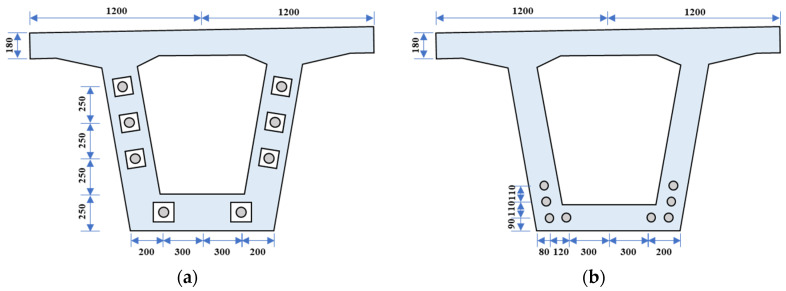
Typical cross-section of the 25 m beam: (**a**) End section (Section A-A); (**b**) Mid-span section (Section B-B).

**Figure 2:**
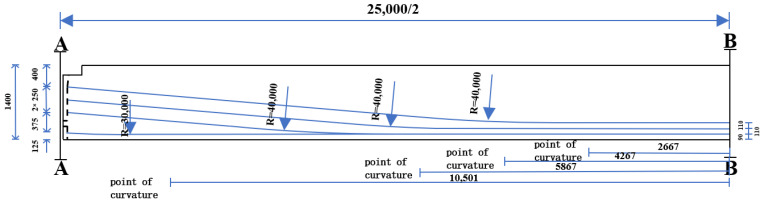
Half-span steel tendon configuration of the 25 m beam. (R is the bend radius of tendon).

The approach bridge of a certain bridge is a 4 × 25 m simply supported-then-continuous prestressed concrete (PC) box girder, designed to bear Highway Class I loads. During its service period, longitudinal and transverse cracks were found on the bottom slab of the girder, and vertical cracks on the web plates. To study the influence of cracks on structural performance and analyze the evolution law of cracks in the box girder after cracking, a 25 m full-scale cast-in-place prestressed concrete box girder was fabricated at the beam yard as the test object. The test girder has a net span of 24.2 m and a height of 1.40 m. Both the main girder and the concrete pavement are made of C50 concrete. The prestressing system adopts low relaxation high-strength prestressed steel strands with a standard tensile strength of 1860 MPa and a diameter of 15.2 mm for a single strand. The general structure of the main girder is shown in Figure 1 and Figure 2.

**Figure 3:**
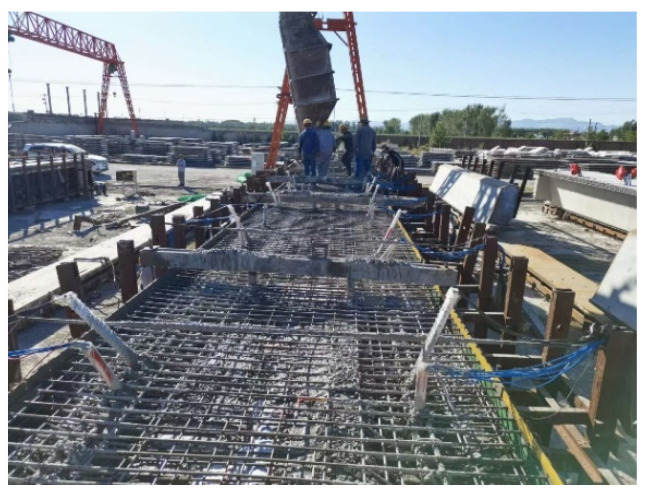
Concrete pouring site.

**Figure 4:**
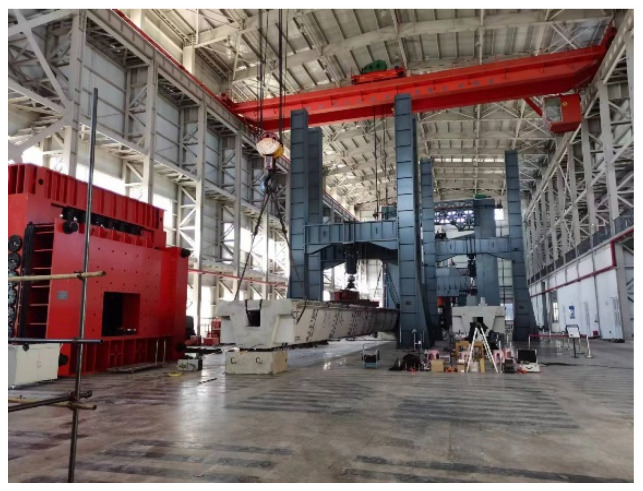
Cracking test site.

The test beams were prefabricated in a precast plant in accordance with standard drawings. Sensors were embedded during fabrication, and strict waterproofing measures were adopted for the embedded sensors. Upon completion and transportation to the test site, displacement transducers and concrete strain gauges were mounted on the concrete surface. The fabrication and test loading setup of the test beams are illustrated in Figure 3 and Figure 4.

### 2.2. Test Setup

**Figure 5:**
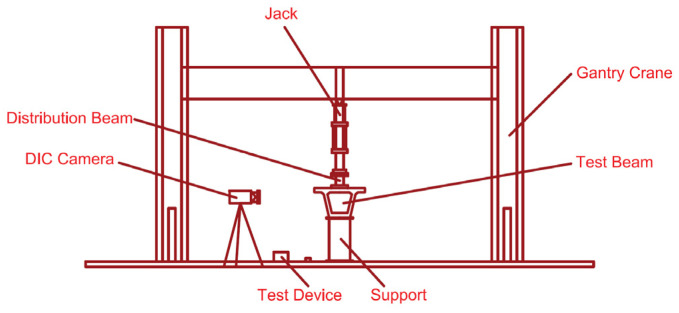
Schematic diagram of test loading.

The test was conducted in the Bridge and Culvert Expansion Building of the Highway Traffic Test Field of the Ministry of Transport. A 3000 kN servo loading system and a 3000 kN ground-anchored reaction frame were adopted as the test devices. The schematic diagram of the test loading is shown in Figure 5.

### 2.3. Crack Observation Layout

The parameters for crack observation include the initial cracking load, crack width, crack length, and typical crack depth. Crack width gauges, tape measures, crack monitors, and the Digital Image Correlation (DIC) technique were employed in the test. Before the test, a grid of 200 mm × 100 mm was drawn on the webs on both sides of the test beam to facilitate the observation of crack propagation and the drawing of crack distribution diagrams. Meanwhile, appropriate-sized random speckles were sprayed on the surface of the left web to enable real-time measurement and data acquisition by cameras during loading. Cracking tests were carried out on the full-scale 25 m test beam, with emphasis on crack propagation during the test. The DIC technique was used to investigate the crack evolution on the left web during loading and unloading, while manual recording and measurement were adopted on the right web to obtain spatial information such as crack length and width.

### 2.4. Loading Procedure

**Figure 6:**
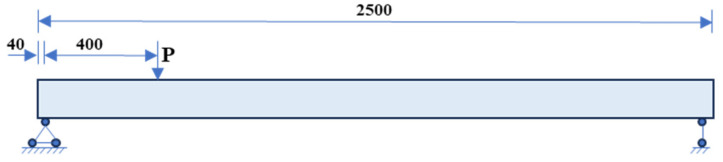
Loading diagram for quarter-point tests (referred to as 1/4 or 3/4 loading for short).

**Figure 7:**
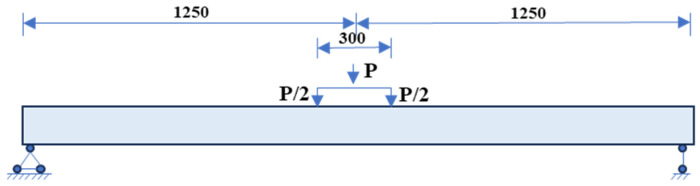
Loading diagram for mid-span test (referred to as 1/2 loading).

Single-point, two-point and three-point loading schemes were adopted in the test. The full-scale beam is 25 m long with a support distance of 0.4 m from the beam ends. The loading positions are set at 1/4 span, 1/2 span and 3/4 span, as detailed in Figure 6 and Figure 7.

Case 1 (Figure 6): 1/4 span loading

This case aims to induce cracks near L/4 and observe the development of inclined cracks. The loading is divided into 19 levels with an increment of 100 kN per level, from 0 up to 1900 kN. At each loading level, the load is held for 10–20 min to measure and record the beam state, cracks, deflection and other parameters. Measurements are also taken when unloading to zero.

Case 2 (Figure 6): 3L/4 span loading

This case is designed to generate cracks near 3L/4 and monitor the cracking process. The loading includes 18 levels with an increment of 100 kN per level, from 0 up to 1800 kN.

Case 3 (Figure 7): 1/2 span loading

This case is used to investigate the crack evolution law after cracks have formed near L/4 and 3L/4. The loading is divided into 17 levels with a 100 kN increment at each level, applying step-by-step and cyclic loading from 0 up to 1700 kN.

## 3. Relationship Between Fractal Dimension and Crack Parameters

In 1975, Mandelbrot proposed the concept of the fractal dimension (FD), which is a key parameter for characterizing fractal analysis. It breaks through the limitation of traditional Euclidean dimensions (integer dimensions) and provides a quantitative analysis tool for the study of concrete damage evolution and crack propagation. The box-counting method is an effective approach among various methods for calculating the fractal dimension of graphics. For research on damage cracks, the calculation formula is as follows:

(1) $$\log N(r)=FD\cdot \log r^{-1}+\ln K$$

where K is a constant; r is the grid size of the basic element, varying as 1/*r^n^* (n = 0, 1, 2, …); and N(r) is the number of boxes required to cover the damaged cracks with grid size r.

To verify the applicability of using the fractal characteristics of crack patterns to evaluate the structural performance of prestressed concrete bridges, the box-counting method was adopted. Grids of different scales were overlaid on the crack maps of the test beams for which the fractal dimension was to be determined, and the number of grids containing cracks, N(r) was calculated. Based on these data, the relationship curve of the apparent cracks of the test beams was plotted.

In the counting process of the box-counting method, a box is counted as 1 regardless of whether one or multiple element dimensions fall within it. It is necessary to calculate the pixel density within each box to represent the probability that the image falls into each box.

(2) $$P_{i}(r)=\frac{N_{i}(r)}{\sum_{i=1}^{N(r)}N_{i}(r)}$$

**Figure 8:**
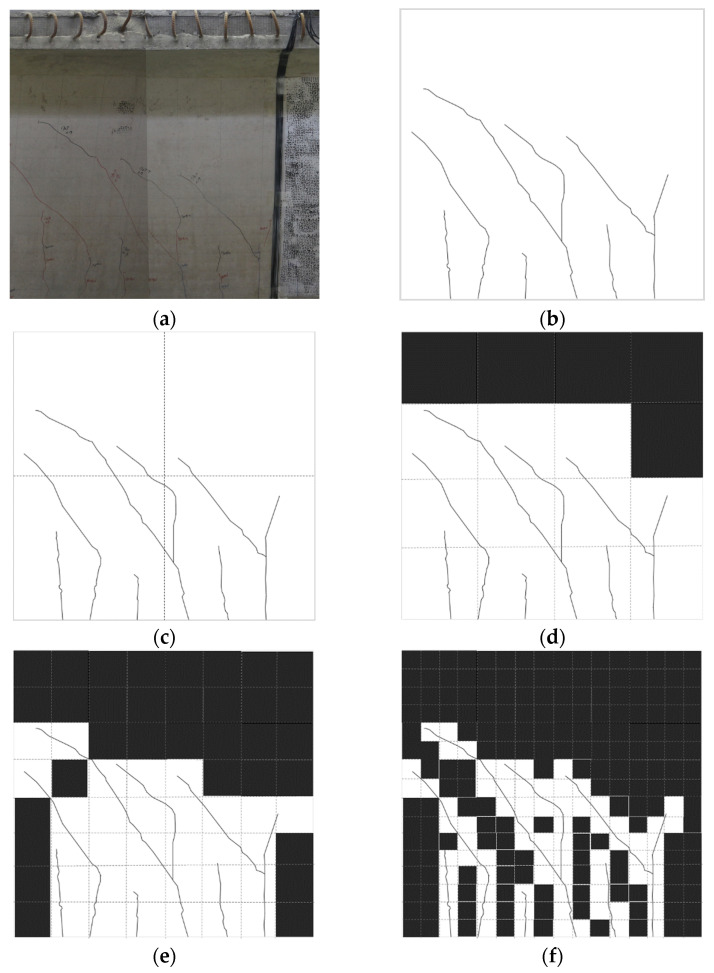
Probabilistic spatial pattern of multifractal analysis for concrete cracking images: (**a**) concrete cracking image; (**b**) crack distribution extraction; (**c**) *r* = 512 pixel; (**d**) *r* = 256 pixel; (**e**) *r* = 128 pixel; (**f**) *r* = 64 pixel.

Based on the local cracking images of the test beam, the calculation procedure of the box-counting method is presented. Figure 8a shows the local cracks on the web of the test beam, with an image pixel size of 1024 × 1024. Suppose the side length is L. First, the crack distribution is extracted from Figure 8a, as shown in Figure 8b. Cracks are then covered by boxes of different scales, i.e., r = L/2, L/4, L/8, L/16, …, as illustrated in Figure 8c–f.

## 4. Development Law of Fractal Dimension

**Figure 9:**
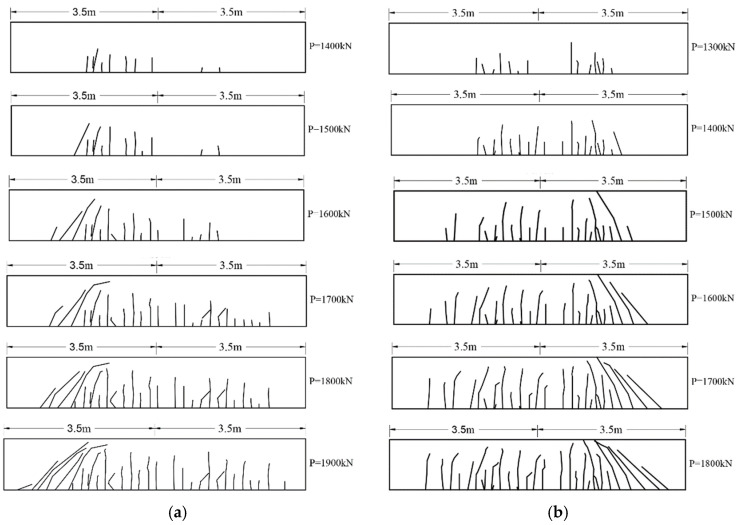

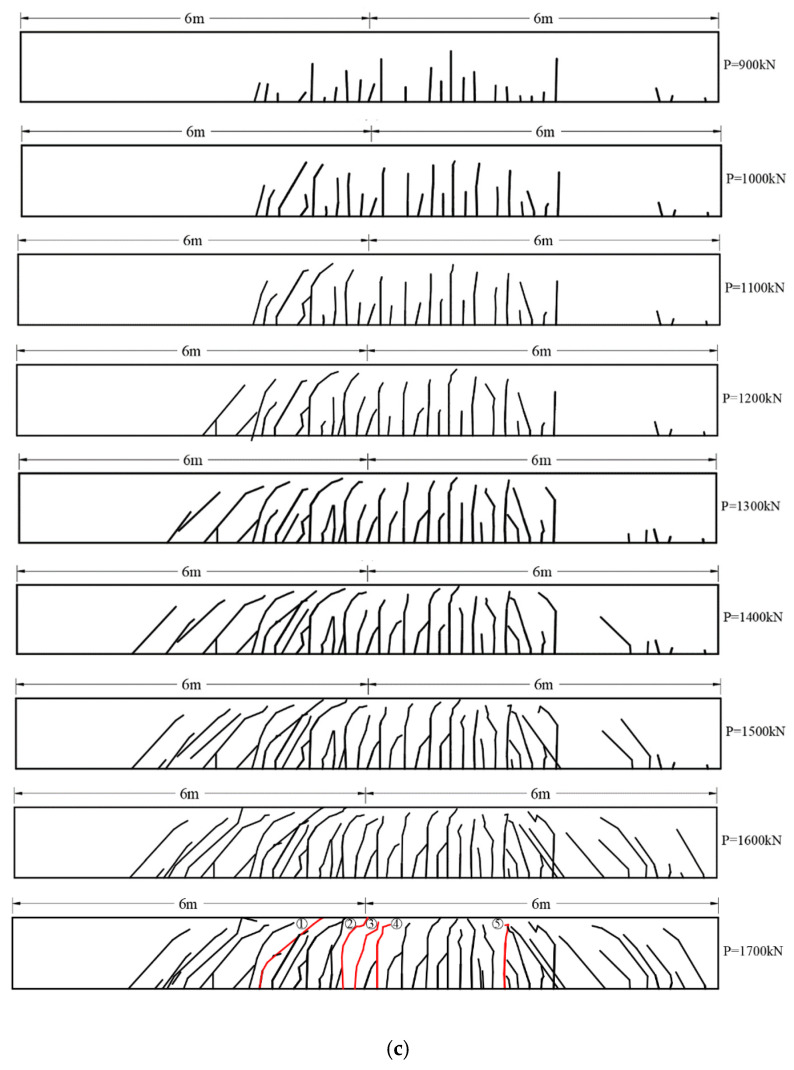
Crack distribution on the right web under various load levels: (**a**) 1/4 Loading; (**b**) 3/4 Loading; (**c**) 1/2 loading (mid-span loading).

**Figure 10:**
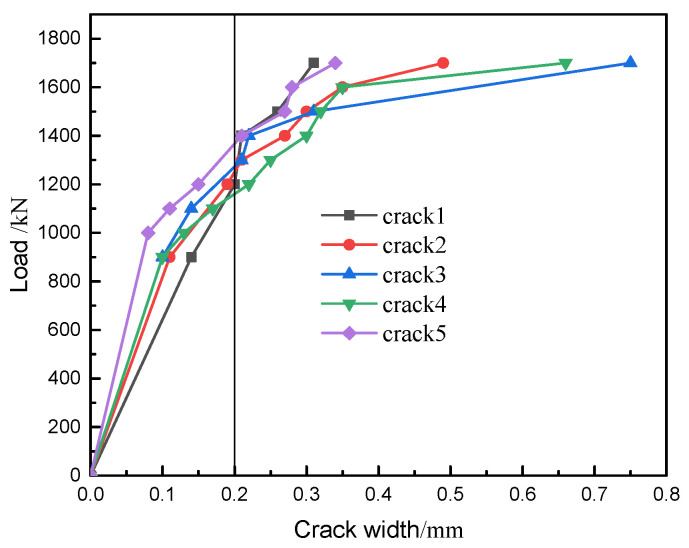
Load-crack width curve.

According to the test data, crack development diagrams at different loading stages were plotted. Figure 9 shows the crack distribution on the right web of the test beam under various load levels. Figure 10 presents the load-crack width curves of typical cracks on both sides of the mid-span when loaded to 1700 kN.

It can be seen from the figures that during the loading process, new cracks continuously appear on the surface of the test beam with the increase in load level, and the crack width gradually widens. According to the actual crack development, the cracks induced by quarter-point loading on the left and right sides are mainly distributed within 3.5 m on both sides of the quarter points, while the cracks caused by mid-span loading are mainly concentrated within 6 m on both sides of the mid-span.

It can also be observed from Figure 9 that the crack distribution is regular, and the crack width growth exhibits a two-stage characteristic: before entering the elasto-plastic stage, the crack width increases relatively slowly; after entering the elasto-plastic stage, the crack width grows rapidly. The widths of Cracks 2, 3 and 4 near the mid-span increase the fastest. When the test beam approaches the ultimate load, the maximum crack width reaches 0.75 mm, as shown in Figure 10.

**Figure 11:**
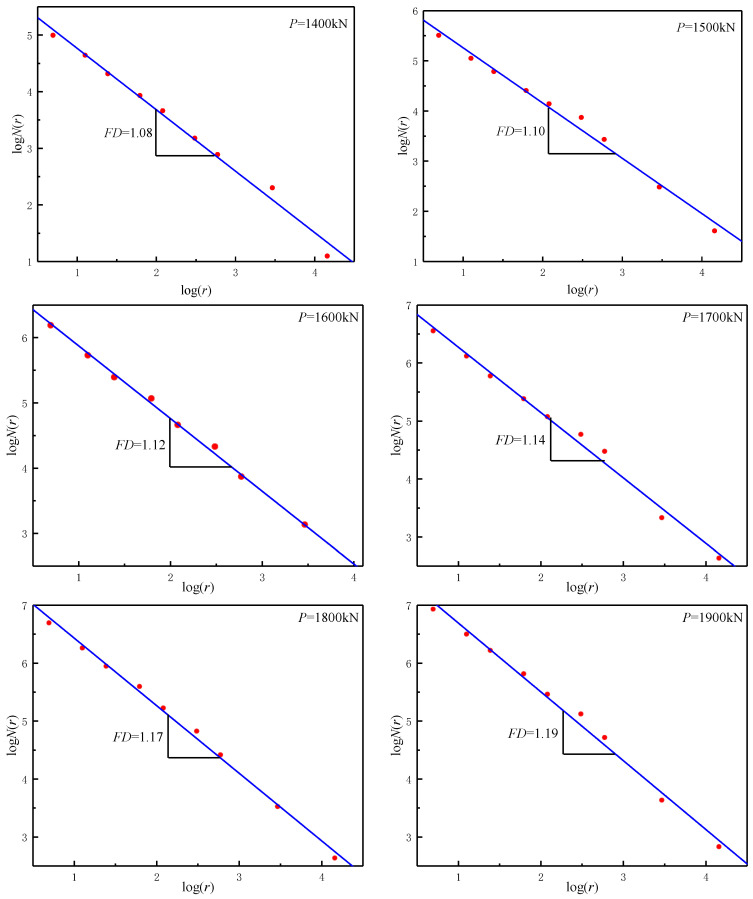
LogN(r)–log(r) relationship curve (1/4 loading). Red dots are data of the test.

**Figure 12:**
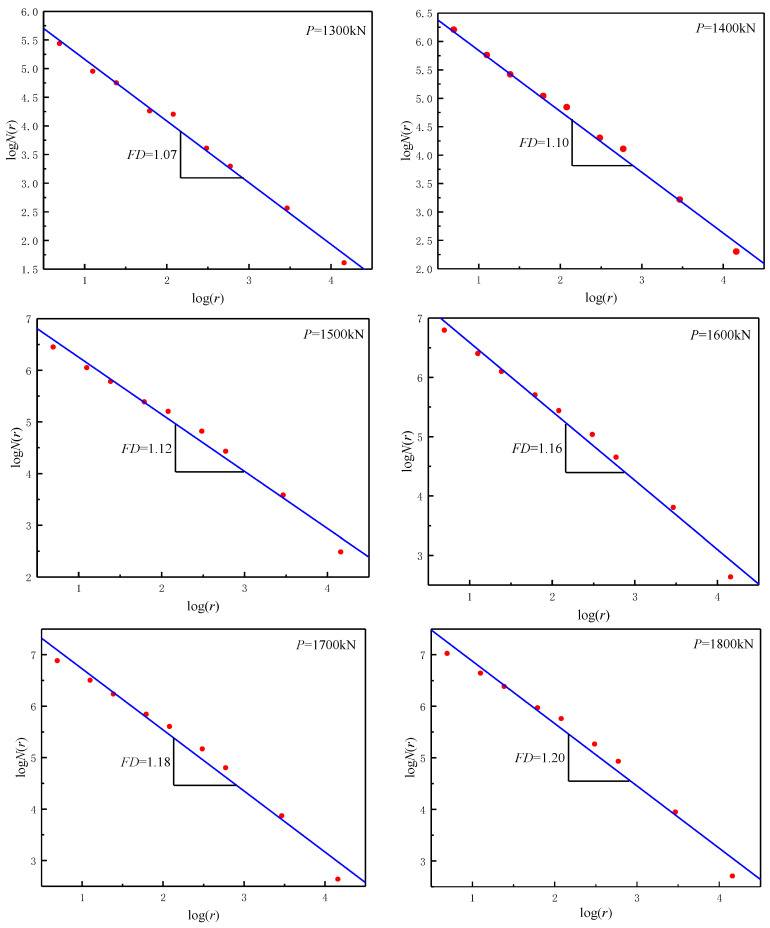
LogN(r)–log(r) relationship curve (3/4 loading). Red dots are data of the test.

**Figure 13:**
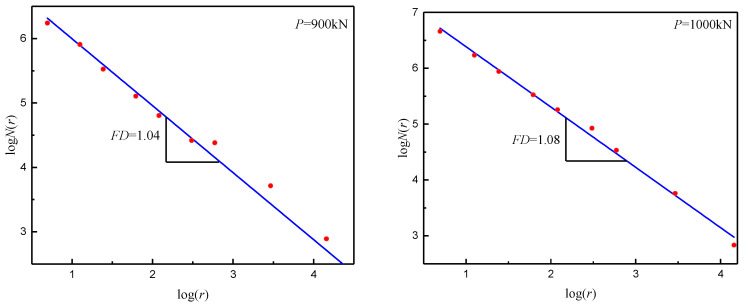

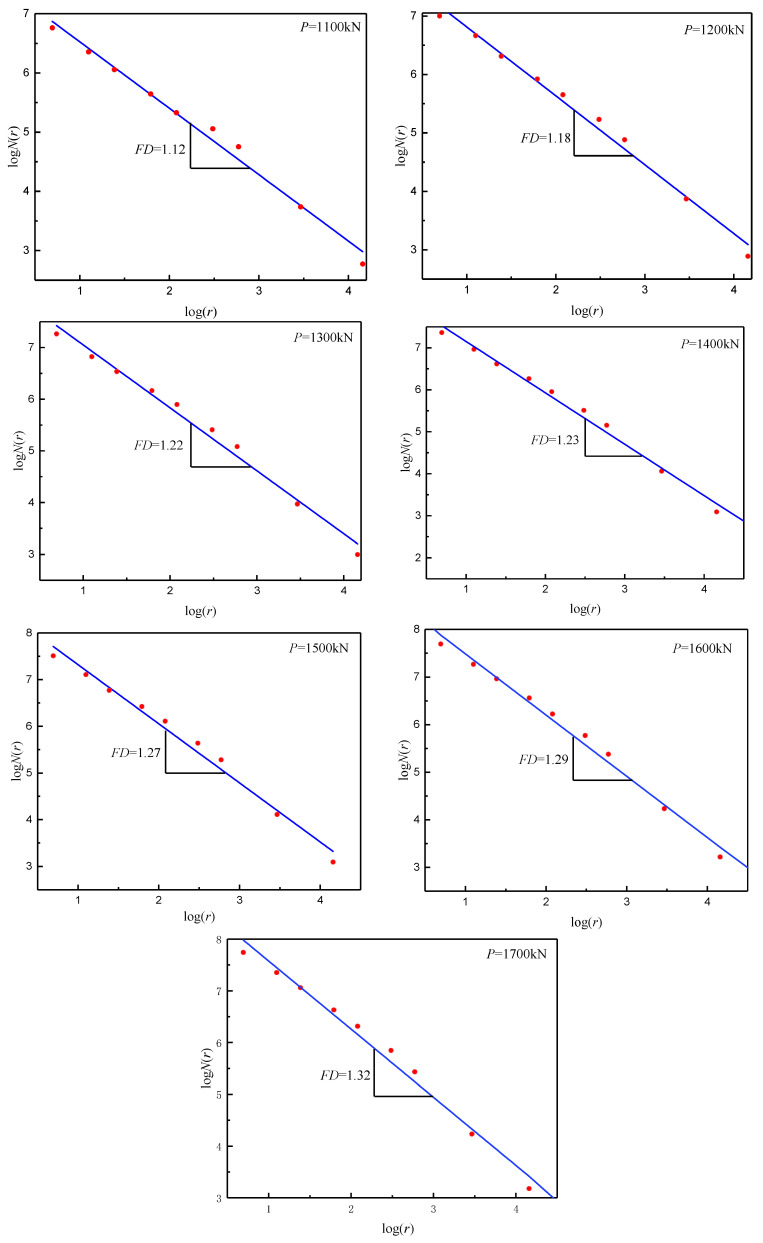
LogN(r)–log(r) relationship curve (1/2 loading). Red dots are data of the test.

Figure 11, Figure 12 and Figure 13 show the logN(r)–log(r) curves of crack distribution in the test beam under quarter-point and mid-span loading.

Under different loading levels, the logN(r) and log(r) values corresponding to a given grid size exhibit a good linear relationship for cracks in the test beam. This indicates that the crack distribution of the test beam satisfies self-similarity and presents statistically significant fractal characteristics, which can reflect the process of crack initiation, propagation and damage evolution, and enable the quantification of crack morphology.

The fractal dimension FD of the beam cracks is obtained from the slope of the logN(r)–log(r) curve fitted by the least squares method. In this test, the fractal dimension of the crack propagation path shows an increasing trend with the loading level. The fractal dimension under quarter-point loading is relatively stable, ranging from 1.07 to 1.20, while that under mid-span loading ranges from 1.04 to 1.32, both of which belong to linear distribution patterns.

**Figure 14:**
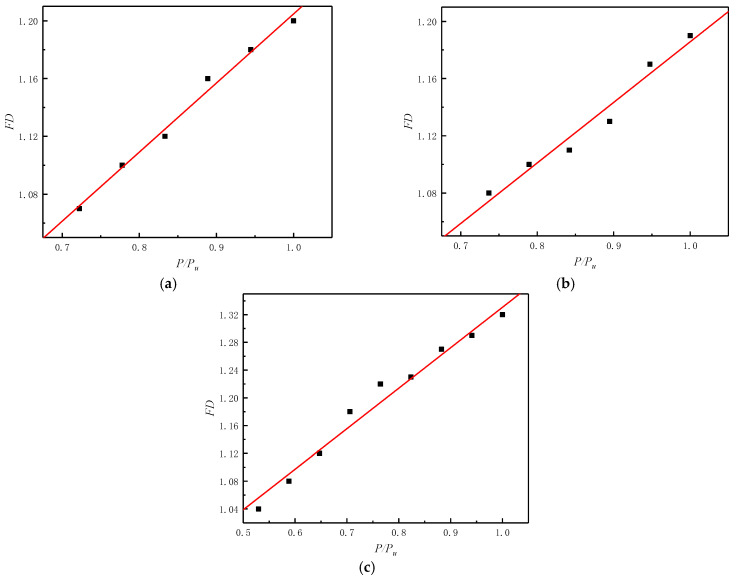
FD–P/Pu relationship curve: (**a**) 1/4 Loading; (**b**) 3/4 Loading; (**c**) 1/2 loading. Black dots are data of the test.

According to the plotted logN(r)–log(r) curves of the test beam, the crack fractal dimension FD under each load level can be fitted. The relationship between the load ratio P/Pu and the corresponding crack fractal dimension FD at each loading stage is plotted in Figure 14.

It can be seen from Figure 14 that the crack fractal dimension FD increases gradually with the increase in the concentrated load applied to the test beam. There is a linear relationship between the crack fractal dimension and the concentrated load, and the correlation coefficient R^2^ of linear regression is all greater than 0.98, indicating a strong correlation between them.

The linear equation is as follows:

(3) $$FD=k(P/P_{u})+B,$$

where k and B are undetermined constants that can be regressed according to the test parameters.

In this test, the values of k under 1/4 loading, 3/4 loading, and 1/2 loading are 0.424, 0.478, and 0.584, respectively, and the values of B are 0.765, 0.726, and 0.747, respectively.

P/Pu is the ratio of the current load P on the member to its ultimate bearing capacity Pu.

It can be seen that the crack propagation process of the concrete beam reflects the fractal dimension-increasing process; that is, the damage evolution process of cracks in the test beam is a dimension-increasing process, and the fractal dimension of the crack distribution increases linearly with the applied load.

**Figure 15:**
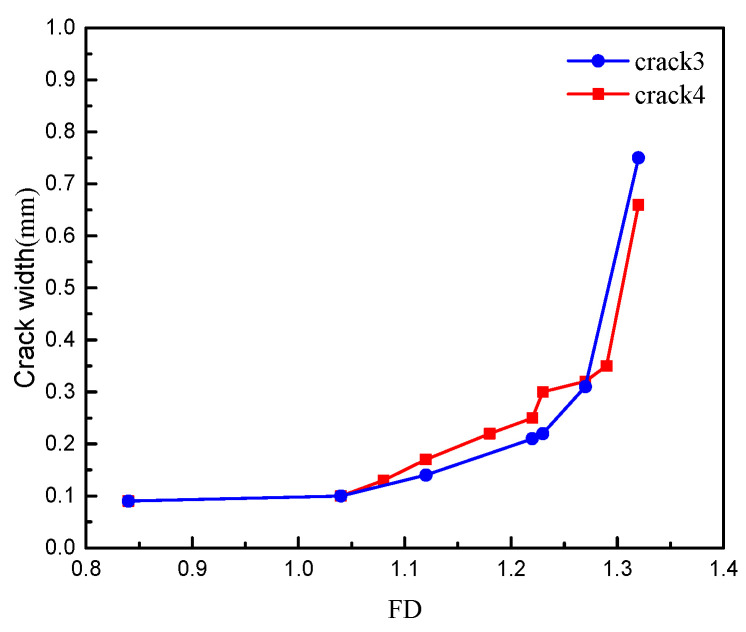
Crack width-FD relationship curve.

**Figure 16:**
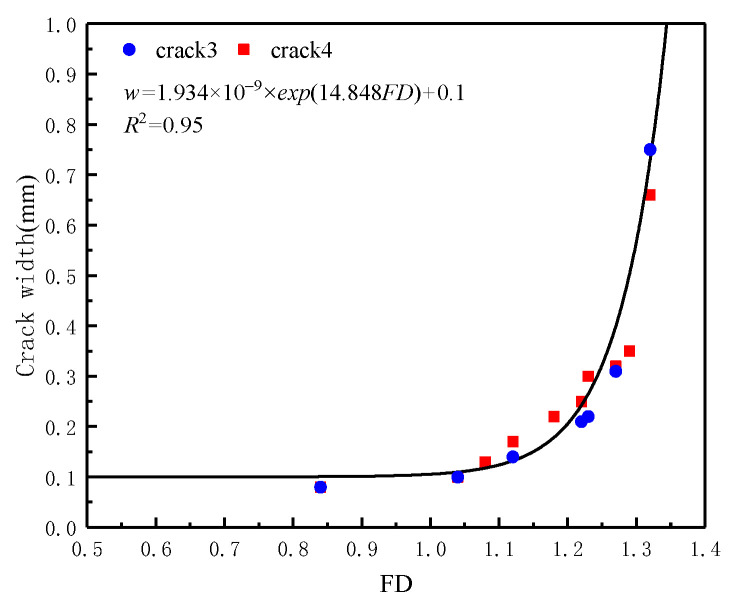
Fitted curve of crack width-FD.

From the above analysis, the fractal dimension can well reflect the development trend of cracks and the change in the structural performance of the test beam. Here, a functional relationship between the maximum crack width and the fractal dimension is established to reflect the influence of the fractal dimension on the maximum crack width. The two widest cracks formed at the mid-span position are selected, and the relationship curve between crack width and fractal dimension FD is plotted, as shown in Figure 15. Based on the scatter plots of these two typical cracks versus fractal dimension, the functional relationship between crack width and fractal dimension is derived, as shown in Figure 16.

Based on the test data of the test beam, the regression equation describing the relationship between crack width and fractal dimension is obtained as follows:

(4) w=mexp(xFD)+d

where w is the maximum mid-span crack width under load; m, x and d are undetermined constants, which are 1.934 × 10^−9^, 14.848 and 0.1, respectively in this test.

Variations in crack width reflect the changes in the structural performance of test beams. Therefore, this paper attempts to establish the correlation between crack width and fractal dimension to characterize the development of crack width based on the surface crack characteristics of test beams. Given the large loading capacity and high safety risks involved in full-scale beam tests, only the width changes in a small number of cracks were monitored in this study, leading to limited experimental data. Nevertheless, the relationship between crack width and fractal dimension has been verified in existing studies [17], and further validation with more full-scale test data is still required.

## 5. Conclusions

This study adopts fractal theory to explore the crack evolution law of prestressed concrete box girders after cracking and analyzes the evolutionary characteristics of fractal features during crack propagation under different loading processes. Firstly, four-point loading was applied to induce structural stress cracks. Subsequently, mid-span loading was carried out to investigate the evolution law of mid-span flexural cracks after flexure-shear cracks emerged. The whole crack evolution process was analyzed via fractal dimension, and the functional relationship between crack width and fractal dimension was established.

The fractal dimension (FD) of cracks in prestressed concrete gradually increases with the rise in concentrated load applied to test beams, showing an obvious linear correlation between fractal dimension and concentrated load. During the formation of shear cracks, the large applied load leads to slow crack propagation, resulting in low sensitivity of fractal dimension. After cracks form under four-point loading, the fractal dimension of flexural cracks generated by subsequent mid-span loading presents favorable sensitivity, which is basically consistent with the test results of reinforced concrete beams reported in existing literature [17].

Furthermore, this study establishes the functional correlation between fractal dimension and crack width to enrich existing structural damage evaluation methods. The results verify that fractal dimension can realize quantitative characterization and dynamic monitoring of surface crack morphology on full-scale prestressed concrete box girders. In future research, fractal theory can be applied to evaluate the durability damage of prestressed concrete beams caused by material deterioration, as well as to assess inclined and vertical cracks in long-span prestressed concrete box girders. In addition, researchers can take crack fractal dimension as a new input feature of machine learning algorithms and combine it with traditional crack parameters, including crack length, width, density and orientation for comprehensive analysis.

Despite promising application prospects, this study still has several limitations. Data collection starts after each loading level stabilizes, and the stability judgment is implemented in accordance with relevant specifications, which is actually affected by the stability of loading oil pressure. Accordingly, certain errors exist in the measurement of crack width and length. Theoretically, the smaller the box size is, the closer the calculated fractal dimension is to the true value. In practice, the box size cannot be infinitely reduced due to the restriction of image pixel resolution.

The research conclusions have certain limitations. Although comparisons and verifications have been conducted with test data of reinforced concrete beams, no comparative analysis with other full-scale prestressed concrete beam tests has been performed. Conclusions drawn based on a single test specimen have certain limitations. Conventional crack parameters, such as crack spacing, have not been fully discussed, which can be regarded as potential research directions in follow-up studies.

Only one full-scale 25 m specimen was adopted in this test. More systematic tests are required in the future. Comprehensive parametric tests should be conducted to clarify the influence laws of concrete strength, prestress level and other factors on fractal dimension. It is also feasible to establish a crack morphology database for various reinforced concrete structures, so as to further broaden the applicability of this method to different structural components and promote its popularization in crack evaluation of long-span prestressed concrete structures.

## Data Availability

The original contributions presented in this study are included in the article. Further inquiries can be directed to the corresponding author.
